# Hypoxia-Inducible Factor-Dependent and Independent Mechanisms Underlying Chemoresistance of Hypoxic Cancer Cells

**DOI:** 10.3390/cancers16091729

**Published:** 2024-04-29

**Authors:** Peter Wai Tik Lee, Lina Rochelle Koseki, Takao Haitani, Hiroshi Harada, Minoru Kobayashi

**Affiliations:** 1 Laboratory of Cancer Cell Biology, Graduate School of Biostudies, Kyoto University, Kyoto 606-8501, Japankoseki.rochelle.35a@st.kyoto-u.ac.jp (L.R.K.);; 2 Department of Genome Repair Dynamics, Radiation Biology Center, Graduate School of Biostudies, Kyoto University, Kyoto 606-8501, Japan; 3 Department of Urology, Graduate School of Medicine, Kyoto University, Kyoto 606-8507, Japan

**Keywords:** hypoxia, chemoresistance, hypoxia-inducible factor (HIF)

## Abstract

**Simple Summary:**

In solid tumors, oxygen concentration varies between different regions. Generally, while oxygen supply is sufficient in the vicinity of blood vessels, the concentration of oxygen gradually drops as the distance from the blood vessel increases. In the regions with low oxygen content (hypoxic regions), cancer cells acquire various malignant properties, like invasiveness, altered metabolism, and therapy resistance, leading to recurrence and poor clinical outcomes of patients. Hypoxia-inducible factor (HIF) is a major regulator of hypoxia responses. Herein, we summarized how tumor hypoxia activates different mechanisms, in both HIF-dependent and HIF-independent manners, and contributes to the acquisition of chemotherapy resistance. We also discussed the involvement of epigenetic regulation in hypoxia-induced chemoresistance, with a specific example of ATAD2 protein degradation inducing drug resistance under hypoxia. Finally, we briefly reviewed some current clinical trials that target HIF and tumor hypoxia for cancer treatment or therapy sensitization.

**Abstract:**

In hypoxic regions of malignant solid tumors, cancer cells acquire resistance to conventional therapies, such as chemotherapy and radiotherapy, causing poor prognosis in patients with cancer. It is widely recognized that some of the key genes behind this are hypoxia-inducible transcription factors, e.g., hypoxia-inducible factor 1 (HIF-1). Since HIF-1 activity is suppressed by two representative 2-oxoglutarate-dependent dioxygenases (2-OGDDs), PHDs (prolyl-4-hydroxylases), and FIH-1 (factor inhibiting hypoxia-inducible factor 1), the inactivation of 2-OGDD has been associated with cancer therapy resistance by the activation of HIF-1. Recent studies have also revealed the importance of hypoxia-responsive mechanisms independent of HIF-1 and its isoforms (collectively, HIFs). In this article, we collate the accumulated knowledge of HIF-1-dependent and independent mechanisms responsible for resistance of hypoxic cancer cells to anticancer drugs and briefly discuss the interplay between hypoxia responses, like EMT and UPR, and chemoresistance. In addition, we introduce a novel HIF-independent mechanism, which is epigenetically mediated by an acetylated histone reader protein, ATAD2, which we recently clarified.

## 1. Brief Introduction

The oxygen microenvironment inside of a solid tumor tissue is highly heterogeneous; the partial oxygen pressure (pO_2_) varies among different regions. In the regions approximately 70~100 μm away from tumor blood vessels, a hypoxic environment exists in which cancer cells are chronically not supplied with sufficient oxygen due to the imbalance between the oxygen consumption by cancer cells and the oxygen supply from blood vessels, which is caused by a low vascular density and an abnormal tumor vessel structure [1]. In addition, the transient occlusion of tortuous and immature tumor blood vessels give rise to an acutely hypoxic environment even in the proximal regions of blood vessels. Previous studies have shown that cancer cells in hypoxic regions demonstrate higher resistance to chemotherapy and radiotherapy; moreover, a higher intratumoral hypoxic fraction has been shown to be correlated with poor prognosis in multiple types of patients with cancer [2,3]. To improve the outcome of cancer treatment, it is necessary to elucidate the molecular mechanisms by which cancer cells acquire resistance to conventional therapies by exploiting the hypoxia response mechanism and to apply these findings to the establishment of novel therapeutic strategies.

In this article, we will first overview the basis of hypoxia-induced therapy resistance due to the physical distance from tumor blood vessels and the resultant decrease in pO_2_, and then we will summarize the knowledge on HIF-dependent molecular mechanisms which has been elucidated from the viewpoints of hypoxia and HIF biology. We will also introduce some HIF-independent mechanisms by which hypoxic cancer cells acquire resistance to chemotherapy. Finally, we will briefly discuss epigenetic gene regulation in relation to hypoxia and present an update on our discovery of an epigenetic mechanism mediated by the HIF-independent function of the ATPase family AAA domain containing 2 (ATAD2) that recognizes acetylated histone H4 [4].

## 2. Hypoxic Microenvironment in Malignant Solid Tumors and Its Association with Therapy Resistance

Cancer cells accessible to sufficient nutrients and oxygen (normoxic tumor cells) actively proliferate around tumor blood vessels in solid tumor tissues, resulting in high oxygen consumption [1,5,6]. In addition, tumor blood vessels are fragile and leakier than normal blood vessels, which causes higher interstitial pressure within tumor tissues and reduced oxygen diffusion. For these reasons, oxygen consumption greatly exceeds oxygen supply within tumor tissues, and this imbalance generates chronically hypoxic regions in the regions approximately 70–100 μm away from tumor blood vessels [1]. Moreover, because tumor blood vessels are tortuous and immature, they often fall into an occluded state, and this induces an acutely hypoxic environment even in the proximal regions of vessels [1].

The high interstitial pressure also results in an insufficient diffusion of anticancer drugs within a tumor tissue [1,7]. Additionally, hypoxia impedes drug delivery as it promotes intratumoral fibrosis. Particularly in pancreatic cancers, there is extensive lysyl oxidase (LOX)-mediated crosslinking of collagen fibers in the tumor stroma, and hypoxia also stimulates stromal cells’ collagen production [8,9,10]. As a result, the highly dense extracellular matrix acts as a physical barrier that impairs drug diffusion from blood vessels.

Moreover, in such hypoxic microenvironments, many chemotherapeutic drugs which mechanistically target actively growing cells, including alkylating agents, platinum-based drugs, and taxanes, do not exhibit full efficacy as hypoxic tumor cells do not actively proliferate and are in a state of cell cycle delay [7,11].

Tumor hypoxia remains a roadblock for not only chemotherapy, but also radiotherapy, especially those involving X-rays and other types of radiation with low linear energy transfer (LET) [6,12,13,14]. The half-life and the amount of cytotoxic reactive oxygen species (ROS) produced by ionizing radiation are increased in the presence of oxygen; conversely, under hypoxic conditions, the lack of oxygen reduces ROS generation/activity and thus hampers the induction of DNA damage by radiation [6,12,15]. It is also known that the ends of DNA double-strand breaks caused by radiation are efficiently oxidized and are less likely to be repaired under normoxia, whereas hypoxia represses such oxidation and results in more transient DNA damage [6,12,15,16]. Thus, it has been confirmed that cancer cells acquire resistance to radiation therapy in hypoxic regions.

In addition to these well-established reasons behind therapy resistance that we have come to understand over the years, the importance of the biological mechanism mediated by hypoxia-responsive genes has also become recognized, as described below.

## 3. HIF-Mediated Mechanisms behind Chemotherapy Resistance of Cancer Cells under Hypoxia

### 3.1. The Molecular Mechanisms behind the Regulation of HIFs’ Activity

HIF, the master regulator of hypoxic responses, is known to promote hypoxia-mediated chemoresistance. HIF functions as a heterodimeric transcription factor, which consists of one α subunit (HIF-1α, HIF-2α, or HIF-3α; hereafter, HIF-αs) and one β subunit (ARNT/HIF-1β), to regulate the transcription of thousands of downstream genes in response to hypoxia stimuli [17]. While the β subunit is stably and constitutively expressed regardless of the oxygen conditions, the expression level of HIF-αs is regulated post-translationally in an oxygen-dependent manner. Under normoxic conditions, prolyl-4-hydroxylase domain (PHD) proteins, which belong to the O_2_/Fe^2+^/2-oxoglutarate (2-OG)-dependent dioxygenases (2-OGDD) superfamily, are catalytically active and hydroxylate HIF-αs at two proline residues located in the oxygen-dependent degradation (ODD) domain (HIF-1α: P^402^ and P^564^; HIF-2α: P^405^ and P^531^) [18,19,20,21,22]. The hydroxylated HIF-αs are then recognized and ubiquitinated by the E3 ubiquitin ligase containing von Hippel–Lindau protein (pVHL) and subsequently degraded by the 26S proteasome machinery [23,24,25,26]. Additionally, in normoxic environments, another 2-OGDD protein, factor inhibiting HIF-1 (FIH-1), can suppress the transactivation activity of HIF; FIH-1 hydroxylates the asparagine-803 residue of HIF-1α (N^847^ of HIF-2α) and thereby obstructs HIFs’ interaction with other transcription co-factors, including p300 and CREB-binding protein (CBP) [27,28,29]. Together, these maintain the low levels of HIF-α proteins as well as HIF activity under normoxia. Contrarily, under hypoxic conditions, the oxygen-dependent PHDs and FIH become inactive, leading to the accumulation of HIF-αs, which interact with HIF-1β and p300/CBP to initiate the transcription of downstream genes. This allows HIFs to promote malignant properties, like angiogenesis [30,31,32,33,34], tumor cell migration/invasion [35,36,37], and metabolic reprogramming to the anaerobic glycolytic pathways [38], under hypoxia.

### 3.2. HIF-Mediated Mechanisms behind Chemotherapy Resistance

**Figure 1:**
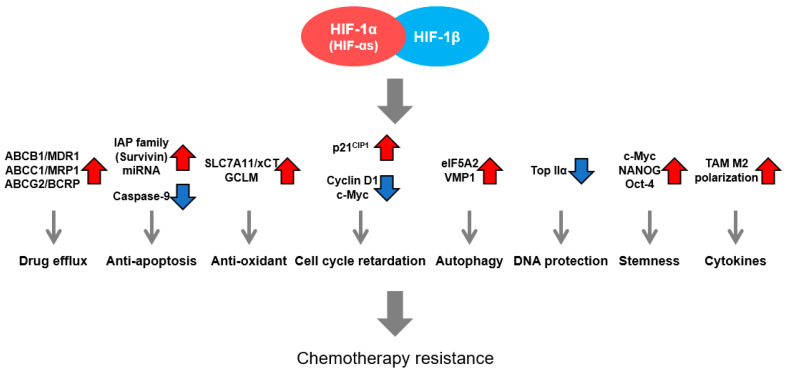
Mechanisms of hypoxia-induced chemotherapy resistance mediated by HIF.

Extensive research has demonstrated the involvement of HIFs in cancer therapy resistance. Below, the major mechanisms through which HIF contributes to chemoresistance will be introduced (Figure 1).

First, HIFs have been reported to promote the chemoresistance of cancer cells under hypoxia by enhancing the expression of ATP Binding Cassette (ABC) transporters, which directly efflux chemotherapeutic agents out of cancer cells [39]. The expression of several ABC transporters is induced under hypoxic conditions in a HIF-1/2-dependent manner, including ABCB1 (Multi-Drug Resistance Protein 1 (MDR1) or P-glycoprotein 1), ABCC1 (Multi-Drug Resistance-Associated Protein 1 (MRP1)), and ABCG2 (Breast Cancer Resistance Protein (BCRP)) [39,40,41,42,43]. In particular, high HIF-1α and MDR1 expression levels were correlated with a higher resistance to 5-FU-based chemotherapy in patients, and sensitivities to various anticancer drugs were restored upon the suppression of HIF-1α/2α [39,40,41,44].

Second, HIF-1 can upregulate the expression of the inhibitors of apoptosis protein (IAP) family in cancer cells to enhance cell viability and drug resistance [45]. It has been reported that HIF-1 induces the expression of a member of the IAP family protein, Survivin, and conversely suppresses the expression of Caspase-9, which is involved in the induction of cell death via the intrinsic apoptosis pathway, thereby enhancing the anti-apoptotic potential of hypoxic cancer cells and leading to resistance to anticancer drugs [45]. In addition, the inhibition of Survivin and HIF-1α in vitro has been shown to sensitize cancer cells to cisplatin treatment by enhancing the activity of the execution caspase, caspase-3 [46]. Recent reports have also elucidated the regulatory role of HIF-1α in microRNA expression to circumvent chemotherapy-induced apoptosis, either by directly targeting factors involved in the caspase-mediated apoptosis pathway or indirectly targeting upstream signaling. For example, under hypoxia, HIF upregulates the expression level of both miR-675-5p, which targets the 3′-UTR region of the caspase-3 mRNA, and miR-27a, which targets the mRNA of APAF1 (apoptotic protease activating factor 1), the major component of apoptosome, and it enhances 5-FU resistance in colorectal cancer cells and paclitaxel resistance in ovarian cancer in vitro. In addition, HIF has been reported to promote docetaxel resistance in triple-negative breast cancer cells by suppressing miR-494 expression to enhance Survivin expression [47]. Furthermore, HIF is known to cause oxaliplatin resistance in colorectal cancer by inducing miR-338-5p expression under hypoxia to promote IL-6 signaling, which, in turn, suppresses apoptosis via the STAT3/BCL2 pathway [48,49,50].

Third, the expressions of SLC7A11/xCT, a cysteine transporter, and Glutamate-Cysteine Ligase Regulatory Subunit (GCLM), an enzyme responsible for glutathione (GSH) synthesis from cysteine, are both induced by chemotherapeutic agents in a HIF-1-dependent manner. This maintains a high level of the reduced form of glutathione to mitigate oxidative stress in cancer cells, rendering higher tolerance to therapeutic drugs [51].

Fourth, the effects of conventional anticancer drugs are attenuated because of the suppression of cancer cell proliferation in the hypoxic microenvironment; congruently, HIF-1 has been shown to negatively regulate cyclin D1 and c-Myc expression and increase p21^CIP1^ protein levels, thereby halting the progression of the cell cycle and negating the effect of chemotherapeutics [52,53]. Intriguingly, it has also been found that chemotherapy-induced senescence, which can be characterized by irreversible cell cycle arrest, is subdued under hypoxia in a HIF-1-dependent manner. This also allows hypoxic cancer cells to survive as well as to retain the potential to proliferate after anticancer drug treatments, indicating that the cell cycle may actually be stringently controlled by HIF under hypoxia via intricate mechanisms, rather than by simple go–stop signals [54].

Fifth, hypoxia can promote autophagy-mediated chemoresistance by the induction of autophagy-triggering proteins, like eIF5A2 and VMP1, both of which are HIF-1 downstream genes [55,56,57]. In addition, HIF has been reported to regulate the key factors involved in various steps of autophagosome formation to promote drug resistance. Beclin-1 (also named ATG6), which is important for the formation of the pre-autophagosomal structure and isolation membrane (autophagy initiation), is induced under hypoxia by the well-known HIF target genes, BNIP3 and FOXO3a, in gemcitabine-resistant bladder cancer and sorafenib-resistant hepatocellular carcinoma, respectively [58,59]. HIF-1 has also been found to upregulate ATG5 expression, which promotes phagophore elongation, and thus induce autophagy-mediated chemoresistance in glioblastoma and lung cancer both in vitro and in vivo [60,61]. Other regulatory mechanisms of hypoxia-induced autophagy, like the PTBP3-ATG12 axis, have also been indicated in chemotherapy-resistant pancreatic cancer in relation to HIF [62]. Of note, the role of autophagy in cancer progression and therapy resistance still remains elusive, as both tumor-suppressing and tumor-promoting mechanisms have been proposed and are highly dependent on the exact conditions of cells and the environment [63,64]. Nonetheless, in the context of hypoxia, a vast majority of reports to date have underpinned the importance of autophagy-mediated chemoresistance.

In addition, HIF has been shown to ameliorate DNA damage induced by chemotherapeutic agents. Breast cancer cells and prostate cancer cells exposed to hypoxia in vitro had reduced levels of topoisomerase IIα, a DNA binding protein essential for DNA-damaging agents to introduce double strand breaks, and such decrease was reversed by the knockdown of HIF-1α. Whilst the detailed mechanism by which HIF regulated topoisomerase IIα expression remains unclear, this study demonstrated that HIF can reduce chemotherapy-induced double strand break and thus causes resistance to etoposide treatment in hypoxic cancer cells [65].

Furthermore, the presence of cancer stem cells (CSCs) in tumors is known to confer chemotherapy resistance, and HIF has been found to foster a CSC enrichment. Intriguingly, HIF-2α increased c-Myc, Oct-4, and Nanog expressions and engendered a stem cell phenotype in breast cancer cells, contributing to Paclitaxel resistance in vitro and in vivo. Conversely, HIF-2α inhibition suppressed stemness and restored sensitivity to Paclitaxel treatment [66,67]. Others, using in vitro and in vivo models, have also demonstrated the involvement of HIF-1 in recruiting tumor-associated macrophages (TAMs) that produce protumor cytokines, e.g., growth/differentiation factor 15 (GDF15), and evoke chemoresistance in a paracrine manner in gastric and colorectal cancer cells. TAMs under low oxygen conditions have also been shown to induce temozolomide resistance in glioblastoma cells by VEGF signaling [68,69,70]. To summarize, numerous research studies have accentuated the role of HIFs in conferring chemotherapy resistance to hypoxic cancer cells via various pathways and mechanisms (Figure 1).

### 3.3. HIF-Related Hypoxia-Responsive Non-Coding RNAs and Chemotherapy Resistance

In the past decade, with advancing RNA sequencing technology, tremendous effort has been devoted to interrogating the regulation of non-coding RNA in response to hypoxia, as well as the clinical relevance of aberrant non-coding RNA expression to therapy resistance [71,72,73,74,75,76]. Hypoxia-responsive non-coding RNAs themselves usually do not directly exhibit biochemical activity for chemoresistance; instead, they provoke changes through the regulation of downstream genes. MicroRNA (miRNA) functions by binding to the complementary sequences in target mRNA, which leads to mRNA degradation or block translation, both resulting in the decreased expression of the downstream target. On the other hand, the two other major types of non-coding RNA, long non-coding RNA (lncRNA) and circular RNA (circRNA), mainly work by sponging miRNAs from their target mRNA and thus interfere with the suppressive effect.

The role of miRNAs in drug resistance has been implicated in a variety of cancers [77,78,79,80,81,82,83,84,85,86]. By regulating the expression of different downstream targets, miRNAs impact a wide range of biological processes that contribute to therapy resistance [78,87,88,89,90,91]. Above, a few examples of HIF-dependent hypoxia-responsive miRNA involved in the regulation of apoptosis have already been introduced. Apart from those pathways, HIF-1α has been shown to induce miR-210-3p expression under hypoxia, which positively regulates TGF-β to enhance in vitro temozolomide resistance in glioma cells [92]. On the other hand, the expression of HIF-1α is also regulated by multiple miRNAs, some of which promote drug resistance [93]. For instance, miR-194-5p was downregulated upon hypoxia treatment, and this increased the expression of its downstream target, HIF-1α, contributing to the hypoxia-induced doxorubicin resistance in A549 non-small-cell lung cancer in vitro [94]. Conversely, miR-301a expression was increased under hypoxia, and it prevented the TAp63-mediated degradation of HIF-1α to induce gemcitabine resistance in MIA-PaCa-2 and BxPC-3 cells [95].

The involvement of lncRNA in therapy resistance has also been widely acknowledged, and there have been extensive studies in the literature oriented towards individual lncRNA or anticancer drugs [71,96,97,98,99,100,101,102,103]. Herein, instead of restating the details of each pathway, we summarized the lncRNAs which have been shown to induce chemoresistance under hypoxic conditions (Table 1). Importantly, whilst many of the existing studies are indicative of the non-coding RNAs being potentially involved in chemoresistance under hypoxia, caution should be taken when interpreting the results, as the functions/regulatory roles of these non-coding RNAs are not necessarily identical under normoxic and hypoxic conditions. Indeed, although there are plenty of studies that have shown the involvement of particular non-coding RNAs in drug resistance or the expression levels of various non-coding RNAs being altered under hypoxia, concrete evidence that comprehensively demonstrates the contribution of non-coding RNA to the chemoresistance of hypoxic cancer cells remains rather limited.

Compared to lncRNA, even less is currently known regarding the role of circRNA in cancer progression and therapy resistance, and there have been very limited reports about hypoxia-responsive circRNAs [104,105,106,107]. To date, only a few hypoxia-responsive circRNAs have been characterized to be relevant to chemoresistance. CircNRIP1 (hsa_circ_0004771) has been shown to be increased by hypoxia in gastric carcinoma cells in vitro, and such increase allowed the sponging of miR-138-5p, which targets the 3′UTR of HIF1A mRNA. This, in turn, upregulated HIF-1α expression and augmented HIF-mediated 5-FU resistance under hypoxia in a glycolysis-dependent manner [108]. Intriguingly, a recent report has demonstrated an alternative regulatory mechanism distinct from miRNA sponging that circRNA induces chemoresistance. In breast cancer cells, hypoxia increased circSTT3A (has_circ_0024760) in a HIF-1α-dependent manner; circSTT3A directly bound with the HSP70 protein to recruit and stabilize PGK1. This promoted the serine-S-adenosylmethionine (SAM)–H3K4 trimethylation axis and enhanced stemness, eventually contributing to doxorubicin resistance in a xenografted mouse model [109].

In addition to the non-coding RNAs expressed in hypoxic cancer cells, the involvement of non-coding RNAs from hypoxia-induced exosomes has also been gradually revealed. For example, in a study of pancreatic cancer, a circRNA microarray identified that circZNF91 is more abundant in exosomes from hypoxic cells. This circRNA sponges miR-23b-3p to enhance SIRT1-HIF-1α signaling in recipient cells and thereby induces gemcitabine resistance in cell culture and xenografted tumor models [110]. In a separate study, it has been shown that HIF-induced exosomal miR-21 from hypoxic CAFs activates the RAS-AKT-ERK pathway and promotes gemcitabine resistance in the recipient pancreatic cancer cells both in vitro and in vivo [111].

Together, these results demonstrate the role of hypoxia-responsive non-coding RNAs, acting either upstream or downstream of HIF, in hypoxia-induced chemoresistance.

### 3.4. HIF, EMT, and Chemotherapy Resistance

Epithelial-mesenchymal transition (EMT) initially describes the process of cell morphology changes wherein cells lose epithelial characteristics and acquire mesenchymal characteristics. However, with an increasing understanding of the molecular basis that governs EMT, it is recognized that a group of transcription factors (EMT-TFs), including SNAIL, SLUG, ZEB, and TWIST, plays a pivotal role in regulating EMT. The expression statuses of EMT-TFs and their downstream targets (EMT markers), rather than morphological changes, essentially became accepted for assessing the occurrence of EMT. Under hypoxia, EMT-TF expression and signaling are mainly upregulated by HIF-mediated transcriptional activation, as many of the EMT-TFs contain HRE in their promoter regions and are direct targets of HIF. In addition, the crosstalk with other pathways (e.g., NF-κB, NOTCH, and β-catenin) which further potentiate HIF-induced EMT has also been demonstrated [112,113,114,115].

In cancer, EMT contributes to disease progression not only by promoting invasion and metastasis, but also by affecting tumorigenesis, metabolic reprogramming, tumor dormancy, stemness, genome stability, etc. [116,117,118]. Moreover, EMT has been shown to be associated with resistance to radiotherapy and chemotherapy and thus may contribute to unfavorable outcomes in patients. The interplay between hypoxia and EMT, as well as that between EMT and chemoresistance, has been vigorously studied and reviewed, so the details will not be reiterated herein [119,120,121,122,123,124,125].

Notably, in order to elucidate the linkage between EMT-TFs/EMT markers and chemoresistance, a vast majority of mechanistic studies utilized in vitro and xenograft models, and patient samples/databases have been used for correlational studies. Compelling evidence comes from two separate studies employing transgenic mouse models. Zheng et al. showed that the abrogation of EMT-TF (SNAIL or TWIST) enhances the sensitivity to the gemcitabine of primary PDAC tumor [126]. And Fischer et al., by distinguishing the epithelial/mesenchymal transcription activity of cells with transgenic fluorescent proteins, showed that cells that have undergone EMT in primary mammary adenocarcinoma are significantly less susceptible to cyclophosphamide treatment [127]. More recently, another group unraveled the mechanism responsible for EMT-associated chemoresistance in primary skin squamous cell carcinoma, showing that EMT cells express the small GTPase RHOJ to regulate nuclear actin dynamics and promote DNA repair, thereby inducing resistance to cisplatin and 5-FU treatment [128].

However, it should be noted that the experiments and analyses in these studies were performed without taking oxygen content into account; so, whether these mechanisms can function identically in hypoxic cells to induce chemoresistance remains to be verified. Also, whilst hypoxia is considered to be an important driver of EMT via HIF activation, studies have shown that treatment with chemotherapeutic agents could trigger EMT and subsequently promote resistance and metastasis in vitro and in vivo [129,130,131,132,133,134,135]. Clarifying the difference between hypoxia/HIF-induced EMT and chemotherapy-induced EMT may help reveal the relevant molecular mechanisms that hypoxic cancer cells exploited to induce EMT-mediated chemoresistance. Indeed, a continuous effort has been made to examine the mechanism of hypoxia contributing to EMT-mediated chemoresistance [136,137,138], and it has been reported that in hepatocellular carcinoma cells and colorectal cancer cells, the in vitro blockade of hypoxia-induced EMT with different small-molecule compounds could potentially attenuate HIF-mediated chemoresistance [139,140].

## 4. HIF-Independent Mechanisms behind Chemotherapy Resistance of Cancer Cells under Hypoxia

**Figure 2:**
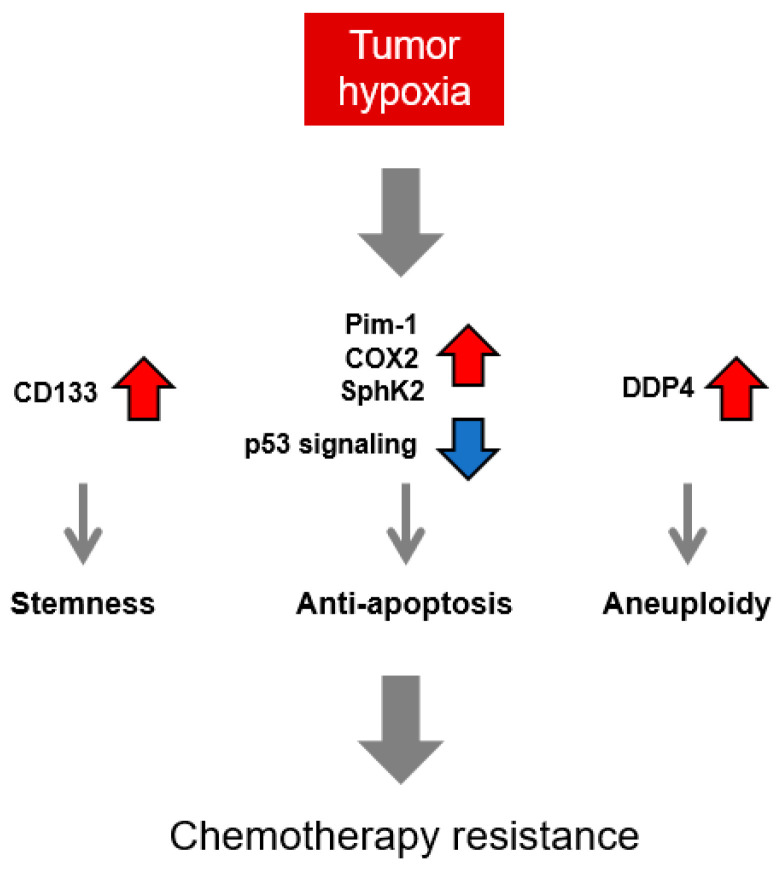
Mechanisms of hypoxia-induced chemotherapy resistance independent of HIF.

Whilst HIF functions as a robust transcription factor regulating multiple downstream targets upon hypoxia and induces the drug resistance of hypoxic cancer cells, other HIF-independent molecular pathways have also been reported to contribute to chemotherapy resistance under hypoxia (Figure 2).

### 4.1. HIF-Independent Mechanisms behind Chemotherapy Resistance

In response to DNA-damaging drugs, hypoxic cancer cells also rely on HIF-independent mechanisms to circumvent apoptosis and thereby develop resistance to chemotherapy. p53 signaling has been reported to affect the drug resistance of U2OS osteosarcoma cells under hypoxia. Treatment with DNA-damaging agents, including cisplatin, etoposide, doxorubicin, etc., can trigger p53 activation by phosphorylation at the Ser-15 residue under normoxia. But the phospho-p53 (Ser-15) level is significantly lowered under hypoxia despite drug treatment, and such a decrease is independent of HIF-1 activity. Correspondingly, the expression levels of p53 downstream targets like NOXA and PUMA are also suppressed under hypoxia after drug treatment, potentially mitigating the pro-apoptotic effect induced by these chemotherapeutic drugs [141]. In other studies using the Hep G2 hepatoma cell, it has been shown that hypoxia decreases p53 expression, resulting in the suppression of the expression of pro-apoptotic protein BAK1, and enhances the JNK-AP-1 pathway, resulting in the reduction in apoptosis upon etoposide treatment [142,143]. Moreover, the expression of the anti-apoptotic factor Pim-1 is enhanced under hypoxia in a HIF-independent manner, leading to decreased activities of caspase-9 and caspase-3 and the stabilization of mitochondrial transmembrane potential. Such suppression of the intrinsic apoptosis pathway results in a higher resistance to cisplatin treatment in hypoxic pancreatic cancer cells, and it was also shown that the forced expression of a dominant-negative form of Pim-1 can sensitize xenografted tumor to chemotherapy in vivo [144]. Other HIF-independent pathways that enhance resistance to chemotherapeutic agents by suppressing apoptotic signals under hypoxia have also been reported in A549 lung adenocarcinoma cells, including the cyclooxygenase-2 (COX-2)–prostaglandin E_2_ (PGE_2_) pathway and the Sphingosine Kinase 2 (SphK2)–Sphingosine-1-Phosphate (S1P)–MAPK pathway [145,146].

Additionally, hypoxia-responsive non-coding RNAs functioning independent of HIF have been shown to contribute to chemoresistance. For example, the hypoxia-inducible lncRNA HIF1A-AS2 regulates HMGA1 expression to suppress p53 family protein activities under hypoxic conditions. This was shown to downregulate Bax expression and promote cisplatin resistance in bladder cancer cells in vitro [147]. Also, circELP3 (hsa_circ_0001785) has been shown to be elevated under hypoxia in a HIF-independent manner, and its depletion by siRNA-mediated knockdown abolished the hypoxia-induced cisplatin resistance in bladder cancer cells in vitro. However, the detailed molecular mechanism like the target of this circRNA was not elucidated [148]. CircUBE2D2 (hsa_circ_0005728) has also been found to promote glycolysis and induce chemoresistance in hepatocellular carcinoma cells under hypoxia; this circRNA was increased in vitro after exposure to mild hypoxia (5% O_2_) and could sponge miR-889-3p to upregulate the expression of LDHA, through which it is suggested to induce sorafenib resistance [149]. Also, in vitro assays have shown that exosomes from hypoxic pancreatic cells have larger amounts of the lncRNA lncROR, and this suppresses the Hippo-YAP pathway in the recipient cells, rendering them gemcitabine-resistant by preventing cell cycle arrest and apoptosis [150]. And the exosomes from hypoxic stromal cells, like cancer-associated fibroblasts (CAFs), contain the hypoxia-responsive lncRNA H19, which activates the DNMT-miR-497 axis and induces paclitaxel resistance in recipient breast cancer cells [151].

CD133 expression has been reported to be upregulated via HIF-dependent and HIF-independent pathways under hypoxia, and both can render resistance against temozolomide and cisplatin to glioblastoma cells [152,153]. Notably, CD133 is commonly used as a surface marker of cancer stem cells. In separate studies using patient-derived samples of colon cancer or rectal cancer, it was found that CD133-positive cells are mainly located in the hypoxic fractions of tumors, but the expression level of CD133 is inversely correlated with that of HIF-1α in patients receiving preoperative chemoradiotherapy. Also, CD133-positive proliferative cells increased after 5-FU chemotherapy, whereas the amount of CD133-negative proliferative population remained unchanged, indicating the potential contribution of CD133 expression to therapy resistance and post-therapy recurrence in a HIF-independent manner [154,155].

Moreover, hypoxia has been reported to cause aneuploidy in Ewing sarcoma via the HIF-independent DDP4/Neuropeptide Y (NPY)–NPY-Y5 Receptor (Y5R)–Rho A axis. These hypoxic polyploid cells were shown to demonstrate higher resistance to doxorubicin treatment than normoxic cells, implying the role of hypoxia-induced polyploidy in chemotherapy failure [156,157,158].

Apart from conventional anticancer agents, hypoxia also induces resistance to targeted chemotherapeutic agents. For instance, Sorafenib, an inhibitor of the RAF-MEK-ERK pathway approved for hepatocellular carcinoma treatment, had significantly lower efficacy in inducing apoptosis under hypoxia due to the hypoxic activation of the compensatory Hippo-Yes-associated Protein (YAP) pathway [159,160]. Together, these findings signify the importance of HIF-independent mechanisms. However, since the molecular mechanisms remain largely unknown, it is critical to unveil them and utilize the acquired information to establish a means to overcome hypoxia-induced therapy resistance. Notably, the mechanisms of hypoxia-induced chemotherapy resistance can usually be triggered by multiple pathways. Those activated by HIF signaling, as detailed in the previous section, may also be induced via HIF-independent pathways to confer chemoresistance under hypoxia. For instance, ABCB1 expression, which facilitates drug efflux, can be enhanced by NRF2 and thus contributes to resistance against cisplatin and doxorubicin in hypoxic MCF7 and Hep G2 cells in vitro, respectively [161,162]. NRF2 has also been shown to upregulate the expression of antioxidant proteins GCLC and GCLM in a HIF-independent manner, thereby attenuating oxidative stress induced by cisplatin treatment under hypoxia [162]. In addition, the induction of NURP1 in hypoxic glioma cells has been shown to promote the KDM3A–TFEB axis to enhance autophagy-mediated temozolomide resistance both in vitro and in vivo [163].

### 4.2. Hypoxia-Associated Proteotoxicity, UPR, and Chemotherapy Resistance

Tumor hypoxia is a hostile microenvironment for cancer cells not only because it imposes oxidative stress and metabolic stress, but also due to the proteotoxicity it inflicts. Post-translational protein folding and isomerization in the endoplasmic reticulum (ER) have been shown to be oxygen-dependent; under hypoxia, protein maturation is impaired, and this induces ER stress as unfolded peptides/misfolded proteins accumulate and aggregate in the ER [164]. In order to resolve ER stress, signaling pathways of the unfolded protein response (UPR) are activated by GRP78 (glucose-regulated protein 78, also named BIP) to boost chaperone activity, induce autophagy, and promote ER-associated degradation (ERAD), all of which are directed towards the refolding and/or clearance of misfolded proteins/aggregates.

Notably, the fate of cells after UPR activation is highly context dependent. Depending on the severity and duration of hypoxia/ER stress, as well as on the status and crosstalk with other molecular responses, different branches of UPR pathways could be induced differently, either leading to the upregulation of the pro-survival gene profile, or when ER stress cannot be tolerated, to the initiation of the apoptosis program [165,166,167].

In general, UPR pathways are augmented under hypoxic conditions to help tumor cell survival and cancer progression. Thus, as a pro-survival mechanism to adapt hypoxia, it seems reasonable that UPR is adopted as yet another tactic by hypoxic cancer cells to withstand chemotherapeutic treatment. Indeed, there have been abundant experimental and clinical data demonstrating the aberrant expression of UPR pathway proteins (e.g., GRP78, XBP1(s), PERK, and ATF4) in various types of cancers, and the molecular mechanism by which UPR induces chemoresistance has also been intensively studied [167,168,169]. But, as mentioned above, the result of UPR activation depends heavily on the cellular context, and it has been pointed out that the temporal order of events (whether hypoxia drives UPR or UPR occurs before the hypoxic response) might also be one of the determinants [170]; hence, whether the findings can be extended to the situation in hypoxic cancer cells requires further examination.

More direct evidence of hypoxia-driven URP inducing chemoresistance came from two in vitro studies. The first study used a hypopharyngeal squamous carcinoma cell line and showed that the knockdown of GRP78, abrogating the hypoxic activation of UPR, leads to the increased expression of Bax (pro-apoptotic) and the reduced expression of Bcl-2 (anti-apoptotic) upon cisplatin treatment [171]. The second study showed that hyperoxia treatment (40% O_2_) can redirect UPR to pro-apoptotic signaling and sensitize glioblastoma cells to temozolomide [172]. In addition, recent results have also started to reveal the underlying molecular mechanisms in further detail; one group reported that hypoxia induces the IRE1α-XBP1(s) arm of URP to enhance 5-FU resistance in colorectal cancer cells, likely by unleashing autophagy suppressed by miR-34a [173].

Of note, although the majority of UPR pathways are often considered HIF-independent, accumulating evidence has shown the opposite [66,174,175,176,177,178]. This certainly adds further complexity to deciphering the interplay between hypoxia response and UPR, as well as the resulting effect on chemoresistance under hypoxia.

In addition to UPR, proteotoxic stress is known to activate heat shock transcription factor 1 (HSF1) as the stress-denatured proteins sequester heat shock proteins (HSPs) like HSP70 and HSP90. The liberated HSF1 can then upregulate the transcription of downstream genes, including HSP genes, which will potentially form a feedback loop. Under hypoxic conditions, it has been shown that the expressions of HSF1, HSP70, and HSP90 are increased, and the phosphorylation of HSF1, which is crucial for its transactivation activity, is also enhanced [179,180]. Indeed, several mechanisms by which HSPs contribute to therapy resistance have been proposed, and studies have also suggested that the overexpression of HSF1 may contribute to chemoresistance [181,182]. In particular, HSF1 has been shown to upregulate the expression of the MDR1 gene, and HSF1 overexpression indeed promoted efflux activity and induced resistance to doxorubicin and paclitaxel in melanoma cells in vitro [183,184]. Nonetheless, whether the HSF1-mediated induction of MDR1 also occurs under hypoxia is untested. Similarly, although there are results showing that heat stress induces the expression of superoxide dismutase (SOD), whose antioxidant activity has been implicated in drug resistance, the involvement of HSF1 as well as the linkage with hypoxia remain unknown [185,186,187,188]. Hence, while there are data indicative of the contribution of HSF1/HSPs, with the current information being fragmentary and the lack of concrete supporting evidence to date, their roles in hypoxic cancer cells acquiring chemoresistance are yet to be confirmed.

Regardless, taken all together, with the involvement of HIF-independent mechanisms being increasingly recognized, it is expected that such knowledge can provide us new opportunities and potential targets for overcoming hypoxia-induced chemotherapy resistance.

## 5. The Mechanisms of Therapy Resistance Acquisition through Hypoxia-Dependent Epigenetic Regulations: The Role of a Histone Acetyl Reader Protein, ATAD2

The hypoxic response of tumor cells is largely regulated by HIF-1, as mentioned above; however, in addition to HIF-1, epigenetic modifications such as chromatin condensation have also been thought to play a crucial role in regulating cell cycles and contribute significantly to the therapeutic resistance of hypoxic cancer cells [189]. A typical example of such epigenetic regulation is histone modification. In particular, the acetylation status of histone, which is mainly regulated by histone acetyltransferase (HAT) and histone deacetylase (HDAC), is known to control global transcription activity. HATs enzymatically facilitate the acetylation of various lysine residues in the N-terminal tail of histones, and this neutralizes the positive charge of lysine side chains, thus weakening the interaction between histones and DNA. This results in the destabilization of nucleosomes and the loosening of chromatin (euchromatinization) so that genomic DNA is more accessible to transcription machineries, thereby activating gene transcription. Conversely, HDACs remove the acetyl group from histone tails, leading to chromatin condensation and the suppression of gene transcription [190].

Indeed, it has been shown that hypoxia enhances HDAC activity in general [191]; two major ubiquitously expressed HDACs, HDAC1 and HDAC2, are reported to be rapidly activated in response to hypoxia [192]. At the chromatin level, hypoxia can induce global chromatin compaction and, correspondingly, decrease the amount of diffuse DNA within the nucleus [193]. Moreover, an ATAC-seq (Assay for Transposase-Accessible Chromatin with sequencing) analysis has indicated that chromatin accessibility to promoter regions was generally lowered upon both moderate and severe hypoxia treatments [194]. These results are in line with the global repression of gene transcription activity, as manifested by the decreases in the total RNA and mRNA synthesis of approximately 40–65% and 20–50%, respectively, under hypoxia [55]. Importantly, such transcription repression under hypoxia was retained even in the absence of HIF-1α or HIF-1β, supporting that the HIF-independent epigenetic regulation of gene expression would also substantially influence hypoxic responses and can thus be targeted for overcoming tumor hypoxia.

The mutation and dysregulation of HAT and HDAC have been found to contribute to the development, malignant progression, and recurrence of cancers and leukemia [4,195]. Particularly, it is known that the aberrant activation of HDAC can lead to chromatin aggregation and induce chemotherapy resistance. For example, the HDAC1/4 expression levels are significantly higher in patients with lung adenocarcinoma who are unresponsive to docetaxel-based chemotherapy, and they are correlated with shorter progression-free survival [196], whereas augmented HDAC2 expression has been shown to cause chromatin remodeling and cisplatin resistance in patient-derived ovarian cancer cells [197]. Therefore, in recent years, a variety of HDAC inhibitors have been developed, with several that have already been approved by the FDA for the treatment of hematological malignancies. Continuous effort is devoted to evaluate their applicability to treat solid tumors as well as to sensitize conventional chemotherapy [198].

Nonetheless, the progress towards clinical application for the treatment of solid tumors is rather limited to date [199,200], as adverse outcomes, which can be exemplified by the low efficacy accompanied by significant toxicity in urothelial cancer and exacerbated metastasis in breast cancer, have also been reported [201,202]. Thus, in order to overcome the anticancer drug resistance of hypoxic cancer cells, it is necessary to identify the key genes involved in chemotherapy resistance through hypoxia-dependent epigenetic regulation.

On top of the acetylation status of histones, which is determined by HATs and HDACs, the importance of histone acetyl readers, containing bromodomains (BRDs) that can recognize acetylated lysine residues, has also recently attracted meticulous attention [203]. A sequence analysis has identified over 40 BRD-containing proteins in the human proteome, and studies have found most of these BRD-containing proteins to be crucial to chromatin remodeling and gene transcription regulation [204]. The aberrant expression of BRD-containing protein has been implicated in inflammatory and autoimmune responses, neurological disorders, as well as hematological malignancies and cancers; thus, these proteins have emerged as attractive therapeutic targets for multiple diseases [205,206,207]. Indeed, approximately 15 different BRD inhibitors have already been used in clinical trials for the treatment of various cancers [208]. Yet, the regulation and function of BRD-containing proteins in the context of tumor hypoxia still remain largely unknown; further studies are therefore important to fully explore their potential as therapeutic targets.

ATAD2 is a protein with a bromodomain that binds to acetylated lysines 5 and 12 of histone H4 (H4K5ac and H4K12ac, respectively), and it has been reported to maintain histone hyperacetylation by preventing HDAC2-mediated deacetylation [209,210]. Recognition of these acetylated histones by ATAD2 has been reported to activate transcription factors such as E2F and Myc that positively regulate cell growth and the cell cycle [211,212].

Given the reports of ATAD2 functioning as a co-regulator of these oncogenic transcription factors, we recently analyzed the function of ATAD2 in the cell cycle progression and proliferation of cancer cells and its role in cancer chemoresistance [4]. ATAD2 protein levels were found to be significantly decreased under severe hypoxia (O_2_ < 0.1%), and this was accompanied by the reduction in acetylated histone H3 lysine 27 (H3K27ac) protein levels. The decrease in acetylated H3K27 was partially but significantly rescued by the forced expression of ATAD2, indicating the involvement of ATAD2 reduction in the global heterochromatinization of genome DNA. An FACS analysis subsequently revealed that severe hypoxic stress significantly decreases the proportion of cells proceeding from the early S phase to the late S phase. This phenomenon was significantly reversed by ATAD2 overexpression. These results indicate that ATAD2 works in cell cycle progression in the S phase and that a decrease in ATAD2 causes cell cycle retardation under severe hypoxia.

**Figure 3:**
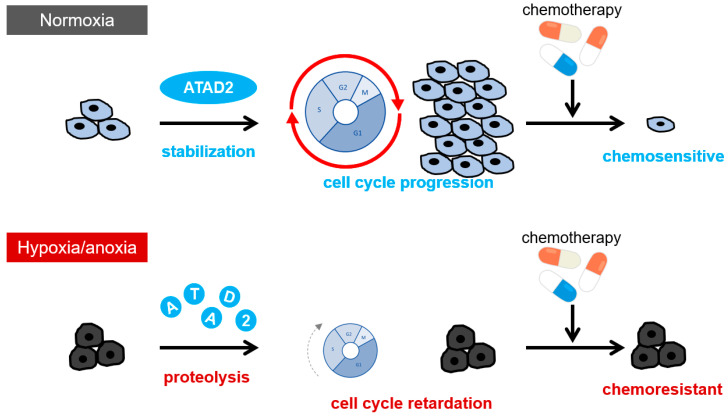
ATAD2 degradation, cell cycle delay, and chemotherapy resistance under hypoxic conditions. Under hypoxic conditions, ATAD2 protein degradation delays the cell cycle from the early to late S phase and induces chemotherapy resistance.

Cell cycle delay caused by ATAD2 reduction was confirmed to contribute to chemotherapy resistance. The knockdown of ATAD2 resulted in the resistance of cells towards a topoisomerase-inhibiting chemotherapeutic agent, camptothecin, even under normoxia. ATAD2-knockout HEK293 cells showed treatment resistance, which was recovered by cell cycle progression forced by ATAD2 reconstitution. Hypoxic stimulation was confirmed to induce resistance to camptothecin, and cells overexpressing ATAD2 were less likely to show resistance to chemotherapy induced by hypoxic stimulation. When administering other common chemotherapeutics, including platinum-based carboplatin and tubulin-stabilizing docetaxel, ATAD2-silenced cells exhibited a higher resistance to these drugs, indicating the generality of this phenomenon. Based on these results, we concluded that hypoxia induces chemotherapy resistance in cancer cells by decreasing the ATAD2 protein levels and delaying cell cycle progression, especially in the early S phase (Figure 3). In addition to this conclusion, our finding that the forced expression of ATAD2 increases the sensitivity of hypoxic cancer cells to anticancer drugs justifies the development of a novel strategy to inhibit the reduction in ATAD2 protein to sensitize hypoxic tumor cells to chemotherapy (Figure 3).

## 6. Oxygen-Dependent Regulatory Mechanism of ATAD2 Expression

How do ATAD2 protein levels decrease in cells exposed to hypoxic stimuli? To answer this question, a strategy to suppress chemotherapy resistance caused by the decreased ATAD2 protein levels under hypoxia should be suggested.

**Figure 4:**
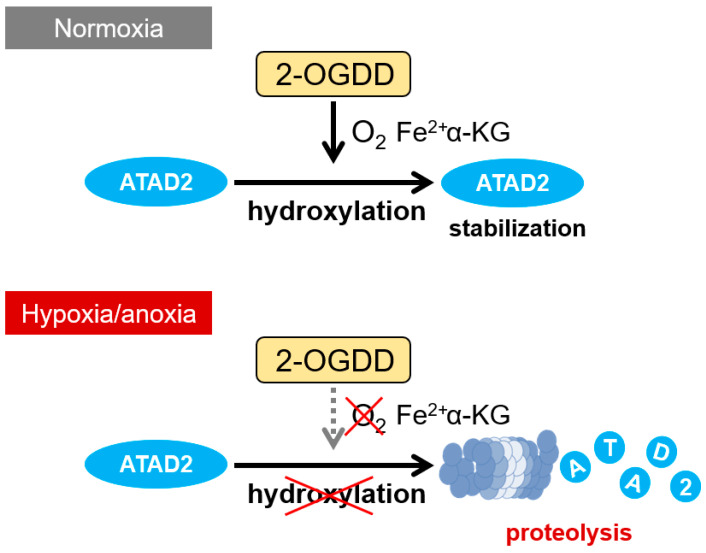
The 2-OGDD-dependent regulation of ATAD2 proteolysis. Under normoxic conditions, the ATAD2 protein is hydroxylated and stabilized by 2-OGDD. On the other hand, under hypoxic conditions, the activity of oxygen-requiring 2-OGDD is reduced, so the hydroxylation of the ATAD2 protein does not occur, resulting in the degradation of the ATAD2 protein by the proteasome pathway. Note: At present, there is also a possibility that a factor regulating ATAD2 stability receives hydroxylation by 2-OGDD.

**Figure 5:**
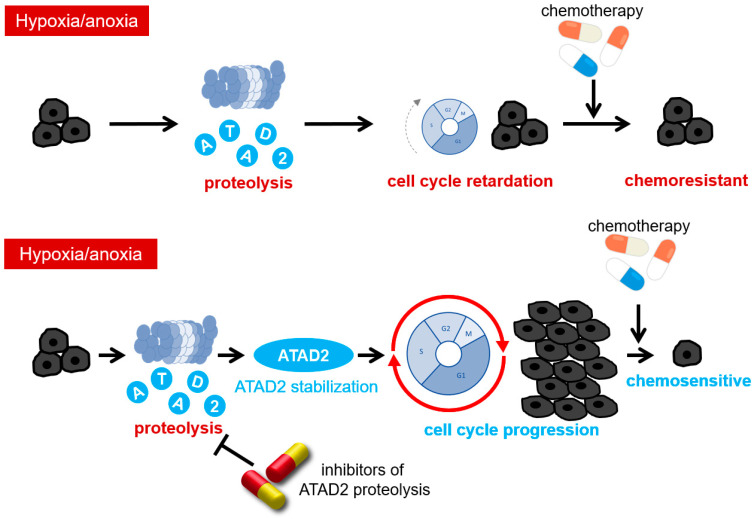
Future perspective: Therapeutic strategies must be developed to overcome the chemoresistance of hypoxic cancer cells via ATAD2 degradation. If the degradation mechanism of the ATAD2 protein can be elucidated and inhibited, a novel therapeutic strategy to overcome the chemotherapy resistance of hypoxic cancer cells can be established.

HIF-1α and its isoforms (HIFs) are stabilized and activated under hypoxia due to the inactivation of PHD and FIH-1, both of which belong to the Fe^2+^/2-OG-dependent dioxygenase (2-OGDD) superfamily. 2-OGDDs require oxygen for their activities, and their importance in hypoxic response has been receiving increasing attention [213]. Intriguingly, the stability of ATAD2 under normoxia was also found to be regulated by a protein belonging to the 2-OGDDs superfamily through the following results [4]: Treatment with an Fe^2+^ chelator, deferoxamine (DFO), or with a 2-OG analogue, dimethyloxalylglycine (DMOG), both of which inactivate 2-OGDDs, resulted in the decrease in ATAD2 protein levels even under normoxic conditions in various types of cells. And a proteasome inhibitor, MG-132, reversed the decrease in ATAD2 protein. Notably, the degradation of ATAD2 protein was observed in cell lines harboring mutant VHL, like clear cell renal cell carcinoma-derived cell lines, RCC4 and 786-O, upon hypoxic treatment, indicating that the regulation of ATAD2 protein was independent of the pVHL–HIF axis and its downstream genes [4]. Together, these results suggest that the chemotherapy resistance of hypoxic cancer cells due to ATAD2 protein degradation is triggered by the inactivation of 2-OGDD and mediated by the proteasome pathway independent of HIFs and pVHL (Figure 4). The further elucidation of this regulatory mechanism, particularly the identification of the responsible 2-OGDD(s), could thus lead to the establishment of new therapeutic strategies to overcome the chemotherapy resistance of hypoxic cancer cells by inhibiting ATAD2 proteolysis under hypoxic conditions and restoring cell cycle delay (Figure 5).

## 7. Summary and Perspectives

In a situation where the microenvironment in tumor tissues is highly heterogeneous and dynamic in time and space, individual cancer cells may acquire resistance to therapy by unique mechanisms. To overcome this problem, it is necessary to analyze how cancer cells adapt to their microenvironments and acquire resistance to therapy, and to develop therapeutic strategies to overcome resistance based on the knowledge obtained. It is known that the mechanisms behind the hypoxia response of cancer cells, which are mainly regulated by HIFs and their downstream genes, play pivotal roles in the chemoresistance of cancer cells under hypoxic conditions.

In the first part of this review, we summarized the major mechanisms triggered by HIF, each with examples of downstream pathways included, showing how HIF induces chemotherapy resistance in hypoxic cancer cells. From these results, it can be acknowledged that the inhibition of HIF would be a promising therapeutic strategy to not only suppress the HIF-induced malignant properties but also to overcome hypoxia-mediated resistance to therapy. Our group has previously reviewed a variety of HIF-targeting anticancer drugs as regards the diverse non-canonical mechanisms regulating HIF expression and activity [5]. Indeed, there has been a continuous effort to put HIF inhibition into clinical trials, with most studies using repurposed drugs that can indirectly inhibit HIF. Currently, almost all trials are Phase I or Phase II studies, and the results are rather limited [214]. In many cases, HIF inhibitors are administered in combination with other anticancer drugs; however, due to drug–drug interactions and differences in cancer types, it is usually difficult to predict clinical outcomes from other trials. For example, Ganetespib, a small-molecule drug that can potentially impede HIF stabilization by targeting HSP90, has been used in a Phase I study together with the antiangiogenic agent ziv-aflibercept to treat patients with advanced carcinoma or sarcoma; however, due to severe toxicity and adverse events, the trial was terminated (ClinicalTrials.gov ID: NCT02192541) [215]. In another Phase I trial targeting patients with a specific type of sarcoma (Malignant Peripheral Nerve Sheath Tumor, MPNST), the combined treatment of Ganetespib and mTOR inhibitor Sirolimus was tolerable, and a partial response was observed in one patient; however, when the study proceeded to Phase II, no responses could be observed despite there being manageable toxicity (ClinicalTrials.gov ID: NCT02008877) [216]. Similarly, in a study using Ganetespib and docetaxel in patients with advanced lung adenocarcinoma, a Phase II trial showed that treatment prolonged progression-free survival and overall survival [217], but the subsequent Phase III trial was terminated, likely because of the lack of supporting data from the interim analysis (ClinicalTrial.gov ID NCT01798485). Regardless, there are still on-going trials (e.g., ClinicalTrials.gov ID: NCT01560416) examining the possibilities of using Ganetespib with other therapeutic agents, as is the case with other HIF inhibitors.

Notably, recent trials have also been exploring the clinical value of HIF-2α inhibition. Promising results from the administration of Belzutifan, a specific oral HIF-2α inhibitor, to patients with VHL germline alteration-associated cancers prompted the approval of its use for the treatment of VHL-associated renal cell carcinoma, central nervous system hemangioblastomas, and pancreatic neuroendocrine tumors [218]. This encouraged trials with a broader application of Belzutifan to other solid tumors (ClinicalTrials.gov ID: NCT02974738), as well as the use of other HIF-2α inhibitors (e.g., ClinicalTrials.gov ID: NCT04895748, NCT05119335, NCT05935748) [219].

With continual clinical trials and studies alongside the enhancing quality of preclinical research, it is envisioned that the HIF inhibitor can be adopted synergistically with other drugs to sensitize chemotherapy treatment in the future.

On the other hand, factors other than HIFs have recently been found to contribute to cellular hypoxic responses as well, and we introduced how some of these HIF-independent mechanisms engender chemoresistance in hypoxic cancer cells. In addition, hypoxia-induced biological responses, like EMT and UPR, involving both HIF-dependent and HIF-independent pathways, have been shown to promote the acquisition of resistance to chemotherapeutic drugs due to the coinciding molecular bases. Although our understanding of their molecular mechanisms has gradually advanced, there remains a lot of unknown parts. The complicated crosstalk therein, along with the resulting context dependence, also adds difficulties to utilize them as therapeutic targets. Hence, basic studies to uncover relevant molecular details would be paramount.

We also briefly discussed the role of hypoxia-responsive non-coding RNAs in promoting chemoresistance. Whilst supportive evidence is accumulating, huge difficulties can still be expected to convert our current knowledge into clinical application, at least in the near future. First, the incomplete understanding of molecular mechanisms would increase the likelihood of unexpected off-target/side effects. Many miRNAs have multiple target mRNAs, and one mRNA may also be regulated by multiple miRNAs; similarly, one lncRNA or circRNA can also regulate multiple miRNAs [220]. Simplistically inhibiting the target RNA molecule may likely result in a disruption to the regulation of other biological processes. Also, most non-coding RNAs originate from the corresponding “parent” genes; targeting non-coding RNA may potentially affect the expressions of the parent genes and other genes containing homologous sequences. Moreover, unlike targeting proteins/enzymes, very little is currently known about the inhibition of specific RNA using small molecules. Although there have been attempts to apply oligonucleotides, sometimes in combination with nanoparticle carriers, most of them were carried out in in vitro studies and did not take oxygen status into account; their applicability to in vivo models or to patients, as well as to hypoxic cancer tissues, is yet to be investigated [221]. Potential obstacles, like unexpected immune responses, should also be considered, and it can be perceived that effective drug delivery, particularly to hypoxic cancer cells, and dosage control would require proper optimization as well. Undoubtedly, the involvement of hypoxia-responsive non-coding RNAs in drug resistance is increasingly being recognized; further studies surveying molecular details and how to utilize them as therapeutic targets in practice would warrant their emerging potential in overcoming chemoresistance in hypoxia.

Given the complexity of the molecular pathways altered under hypoxic conditions, other therapeutic agents have also been tested/developed in an attempt to annihilate hypoxic cancer cells from alternative approaches. For example, metformin and atovaquone are two inhibitors of the mitochondrial electron transport chain and are expected to alleviate hypoxia by reducing cellular oxygen consumption; trials evaluating their oxygenating effect in sensitizing radiotherapy and chemotherapy have been launched (ClinicalTrials.gov ID: NCT04275713, NCT02628080, NCT04648033). Moreover, there were also some hypoxia-activated prodrugs in trials, like CP-506 with carboplatin for triple-negative breast cancer and ovarian cancer (ClinicalTrials.gov ID: NCT04954599), and evofosfamide (TH-302) for recurrent bevacizumab (anti-VEGF antibody)-refractory glioblastoma [222].

Finally, in the latter part of this review, we focused on the contribution of hypoxia-dependent epigenetic regulation and reported a specific example wherein the results demonstrate that the therapeutic resistance of hypoxic cancer cells can be induced by cell cycle retardation because of the degradation of the ATAD2 protein in a 2-OGDD-dependent but HIF-independent manner. Based on this discovery, we opened the potential to develop new therapeutic strategies to overcome the chemotherapy resistance of hypoxic cancer cells by inhibiting ATAD2 degradation under hypoxic conditions and restoring cell cycle delay. While further efforts will be needed to uncover the detailed molecular mechanism and identify the exact 2-OGDD(s) involved, these results exemplify the importance of 2-OGDD proteins, apart from regulating the HIF pathway, in the hypoxic response, shedding light on their potential role in combating the chemotherapy resistance of hypoxic cancer cells.

**Table 1:** List of long non-coding RNAs contributing to chemoresistance under hypoxia in relation to HIF.

	Name	Target/Effect	Resistance	Cancer Type, Model	Ref.
HIF downstream	LINC03000-201 (lncMat2B, ENST00000486913.3)	↓ ROS production ↓ DNA damage	Cisplatin	Breast cancer, in vitro	[223]
	PVT1	miR-140-3p/ATG5 ↓ autophagy	Cisplatin	Lung cancer, in vitro and in vivo	[60]
	lncRNA-CBSLR	YTHDF2/CBS ↓ ferroptosis	Cisplatin	Gastric cancer, in vivo	[224]
	ANRIL	miR-328/ABCG2, MDR1	Cisplatin	Retinoblastoma, in vitro	[225]
	LUCAT1	Interaction with PTBP1 ↓ DNA damage	5-Fluorouracil, Camptothecin, Doxorubicin and Oxaliplatin	Colorectal cancer, in vitro, in vivo, and patient cohorts	[226]
	NLUCAT1 (HIF-2α-dependent)	↓ ROS production	Cisplatin	Lung adenocarcinoma, in vitro	[227]
HIF upstream	PVT1	miR-194-5p/HIF1A ↑ proliferation	Cisplatin	Oral SCC, in vitro	[228]
	HIF1A-AS1	Interaction with YB1 ↑ HIF-1α (positive feedback) ↑ glycolysis	Gemcitabine	Pancreatic cancer, in vitro, in vivo, and patient cohorts	[229]
	HIF1A-AS2	↑ HIF-1α ↑ autophagy	Doxorubicin	Small cell lung cancer, in vitro	[230]
	NORAD	miR-495-3p/HIF-1α ↑ vasculogenic mimicry	5-Fluorouracil	Colorectal cancer, in vitro	[231]
Potentially HIF-dependent	HOTAIR	miR-1277-5p/ZEB1 ↑ EMT	Oxaliplatin	Colorectal cancer, in vitro and in vivo	[232]
	lncRNA-EMS	miR-758-3p/WTAP	Cisplatin	Esophageal cancer, in vitro and in vivo	[233]
